# Selective effects of non-thermal atmospheric plasma on triple-negative breast normal and carcinoma cells through different cell signaling pathways

**DOI:** 10.1038/s41598-017-08792-3

**Published:** 2017-08-11

**Authors:** Yuan Liu, Sheng Tan, Hao Zhang, Xiangjun Kong, Lili Ding, Jie Shen, Yan Lan, Cheng Cheng, Tao Zhu, Weidong Xia

**Affiliations:** 1 School of Life Science, University of Science and Technology of China, Hefei, Anhui 230027 China; 2 The CAS Key Laboratory of Innate Immunity and Chronic Disease, School of Life Sciences, University of Science and Technology of China, Hefei, Anhui 230027 China; 3 Hefei National Laboratory for Physical Sciences at Microscale, Hefei, Anhui 230027 China; 4 Institute of Plasma Physics, Chinese Academy of Sciences, P. O. Box 1126, Hefei, 230031 P. R. China

## Abstract

Non-thermal atmospheric plasma (NTP) has shown its selective anticancer effects in many types of tumors *in vitro* and one of the main mechanisms is that the different increase of intracellular ROS in cancer and homologous normal cells. In this study, we report that NTP treatment reduces the proliferation in triple negative breast cancer (TNBC) and normal cell lines. Simultaneously, STAT3 pathway is inhibited by NTP effects. However, it is observed that normal cells MCF10A are more sensitive to ROS toxicity induced by NTP than cancer cells MDA-MB-231. When 5 mM of ROS inhibitor N-acetyl cysteine (NAC) is employed in NTP treatments, the proliferation of normal breast cells MCF10A recovers. Meanwhile, NTP effects remain significant inhibition of MDA-MB-231 cells. Our results further reveal that NTP can induce apoptosis in MDA-MB-231 cells through inhibiting interleukin-6 receptor (IL-6R) pathway. Moreover, the mechanism of NTP anti-cancer selectivity relates to constantly HER2/Akt activation induced by NTP especially in MCF10A cells but not in MDA-MB-231 cells. Therefore, these two different cell signaling pathways induced by NTP treatments in TNBC and homologous normal cells make NTP becoming a potential tool in future therapy.

## Introduction

Breast cancer is the cause of 23% of the total cancer cases and 14% of cancer deaths, and is the most frequently diagnosed cancers that causes the prominent number cancer death among females^1^. Breast cancer subtypes are ordinarily identified by the presence of estrogen receptor (ER), progesterone receptor (PR), and human epidermal growth factor receptor type 2 (HER2). Triple-negative (TN) patients lack all three. While comparing to other breast cancer subtypes, triple-negative breast cancers (TNBC) are higher frequency and are more aggressive with high potential to metastasize and more lack of targeted treatment options. Chemotherapy is currently the only adjunctive therapy^2^; although triple-negative patients, when regarded as a group, have a worse long-term survival after chemotherapy than patients with other breast cancer subtypes^3, 4^. Toxicity and side effects come along with chemotherapy, while obtained from chemo resistance tumor cells can still maintain viability after chemotherapy^5^. For many women, benefit of chemotherapy is uncertain. Therefore establishing new therapies with little side effects for TNBC is imminent.

Physical plasmas are composed of reactive atoms, molecules, ions and radicals. While non-thermal plasma can allow strong non-equilibrium chemistry because of its ions and neutral species remains cold^6^. Also NTP can produce a mixture of highly-reactive chemical species which play important roles in cell processes^7^. Reactive oxygen and nitrogen species (RONS) induced by NTP that enter adjacent aqueous liquid phases are the key mechanism known to react with important biomolecules^8^. At present, NTP has become as a novel tool in some biomedical areas, such as sterilization, antifungal treatments, blood coagulation, dental care, wound healing and cosmetics targeted cell/tissue removal^9, 10^. NTP treatment has been proven effectively in inducing apoptosis *in vitro* of various tumor types and inhibiting tumor growth *in vivo* ^10^. Moreover, NTP effects on cells were dose dependent that low doses of plasma can stop cancer cells proliferation and high doses result in cell apoptosis and necrosis^11^.

Results of current studies show that NTP can induce apoptosis *in vitro* in various cancer types, from lung cancer, glioblastoma, prostate cancer, melanoma and cervical cancer to breast cancer^12–17^. Previous studies suggest that RONS generated by NTP plays an important role in cell death. In lung cancer, non-thermal plasma induced apoptotic cell death through mitochondrial damage by generating RONS in culture media^18^. In addition, cold atmospheric plasma can induce more intracellular RONS in A549 cells than normal cells^19^. ROS formation in cell culture induces high levels of DNA damage after plasma exposure, and leads to necrosis both in primary prostate epithelial cells and prostate cancer cells^14^. In cervical cancer Hela cells, H_2_O_2_ in plasma-treated medium is the main course of inactivation of cell viability^20^. Earlier study also shows that cold plasma can significantly reduce tumor size through deregulating ROS related genes^21^.

Recent researches are tending to focus on revealing the molecular mechanisms underlying the changes of cancer cells activity by non-thermal plasma. For example, NTP can produce exogenous ROS in cell medium that act as the elementary active species which induce cancer cell death through activating DNA damage^22^. In head and neck cancer, NTP induced apoptotic cell death by involving in MAPK-mediated mitochondrial ROS signaling pathways^23^. Inhibition of the migration and invasion in HeLa cells was induced by NTP via significant depressing MMP-9 expression in ERK1/2 and JNK signaling pathways^24^. NTP also led to sub-G1 arrest in oral cavity squamous cell carcinoma cells and the arrest was related with DNA damage and the ATM/p53 signaling pathways^25^. Furthermore, in the human glioma cell line U373MG, ROS-, JNK- and caspase-independent mechanism of cell death by NTP treatment^26^.

The rise of ROS induced by NTP treatment *in vitro* is the main cause of the cell death. It was reported that hydrogen peroxide (H_2_O_2_) generated by plasma and concentration-matched H_2_O_2_ both resulted in significant rise of intracellular ROS in T helper cells and monocytes, and reduction of cell growth^27^. Former researchers report that air plasma jet treatment induced mitochondrial-mediated apoptosis in HeLa cells was mainly caused by ROS generation^28^. Accumulation of ROS and RNS in HepG2 cells under NTP treatment was reported, because of these effects endoplasmic reticulum stress mediated apoptosis and cellular dysfunction were observed^29^. The exposure of A549 cells to plasma-activated medium (PAM) significantly induced the generation of intracellular ROS and causes caspase-independent apoptosis through involving the mitochondrial–nuclear network^30^. PAM effects on the proliferation inhibition and DNA damage in HCT-116 multicellular tumor spheroid were also reported, while H_2_O_2_ induced by PAM plays an important role in observed genotoxic effect^31^. H_2_O_2_ in PAM and/or •OH generated in association with iron ions also involve in the mitochondrial-nuclear network and result in A549 cell injury^32^. For the application of plasma-stimulated medium (PSM) in future cancer therapy, utilizing stabilizing strategies like storing PSM at 8 °C or −25 °C and adding 3-Nitro-L-tyrosine into DMEM could weaken the degradation plasma-originated reactive species^33^. Recent researchers reported that enhanced level of ROS in U937 cells induced by plasma treatment caused a compromised redox status obvious from reduction of GSH/GSSG ratios, rise of NADP^+^/NADPH levels and total endogenous antioxidant activity decreasing^34^.

NTP selective effects on carcinoma and normal cells were reported and were classified into three groups according to NTP effects on cancer cells: strong selectivity, weak selectivity, and negative selectivity^35^. It was observed that intracellular ROS rising significantly in cancer cells A549 and increased slightly in normal cells HEK293T and BEAS-2B after plasma exposure. Meanwhile, cellular viability of HEK293T and BEAS-2B cells did not show significant change compared to A549 cells^36^. NTP shows strong and weak selective anticancer capacity to most studied cancer cell lines, while only papilloma cells and prostate cancer cells show high resistance to NTP. It was point out that differentiation between normal cells and cancer cells may have correlation with the NTP selectivity^35^. However, NTP effects on the death of normal cells were often observed in some cases and as long as the cells were under NTP treatment, this phenomenon could not be avoided. For example, plasma-induced necrosis and autophagy in primary prostate epithelial cells were recorded^14^. Also, apoptotic cell death in mammalian breast epithelial cells induced by NTP treatment was reported^37^. In addition, atmospheric pressure plasma was observed to suppress cell proliferation of human aortic endothelial cells^38^. ROS generated by atmospheric plasma may cause the death of normal cell in these cases, because of its proverbial damage to DNA^39^. Furthermore, cancer cells from different tissues have quite distinct response to NTP treatment, so it is more meaningful and important to study NTP selective anticancer capacity on normal cells and homologous cancer cells^35^.

Here we mainly paid attention in the NTP effects on breast carcinoma and normal cells. Previous research reported that migration and invasion of breast carcinoma MDA-MB-231 cells can be significantly inhibited by cold atmospheric plasma treatments^40^. Also breast cancer cells (MCF-7 and MDA-MB-231) with quite distinct range of cell confluences showed different response to the same NTP treatment time dose^41^. Additionally, non-thermal plasma treatment can induce apoptosis and the formation of single-stranded DNA breaks of mammalian breast epithelial cells MCF10A^37^ and many cancer cells due to the formation of intracellular ROS. It was reported that U373MG cells demonstrate a higher resistance against H_2_O_2_, but NTP treatments still show significantly killing effects on U373MG cells^26^. However, NTP selective anticancer capacity on TNBC cells has not been reported and the mechanism of NTP effects on breast cancer cells and homologous normal cells is unclear. In this study, we intend to find out the signaling pathways involved in NTP selective killing effects and avoid ROS toxicity induced by NTP to TN breast normal cells MCF10A. We investigated the cell viability and intracellular ROS change in MDA-MB-453, MDA-MB-231 and MCF10A cells under NTP treatments. Moreover, in this work we reveal the different signaling pathways regulated in MDA-MB-231 and MCF10A cells under NTP treatment.

## Results

### NTP treatment reduces the proliferation of both TN breast normal and carcinoma cell lines

**Figure 1:**
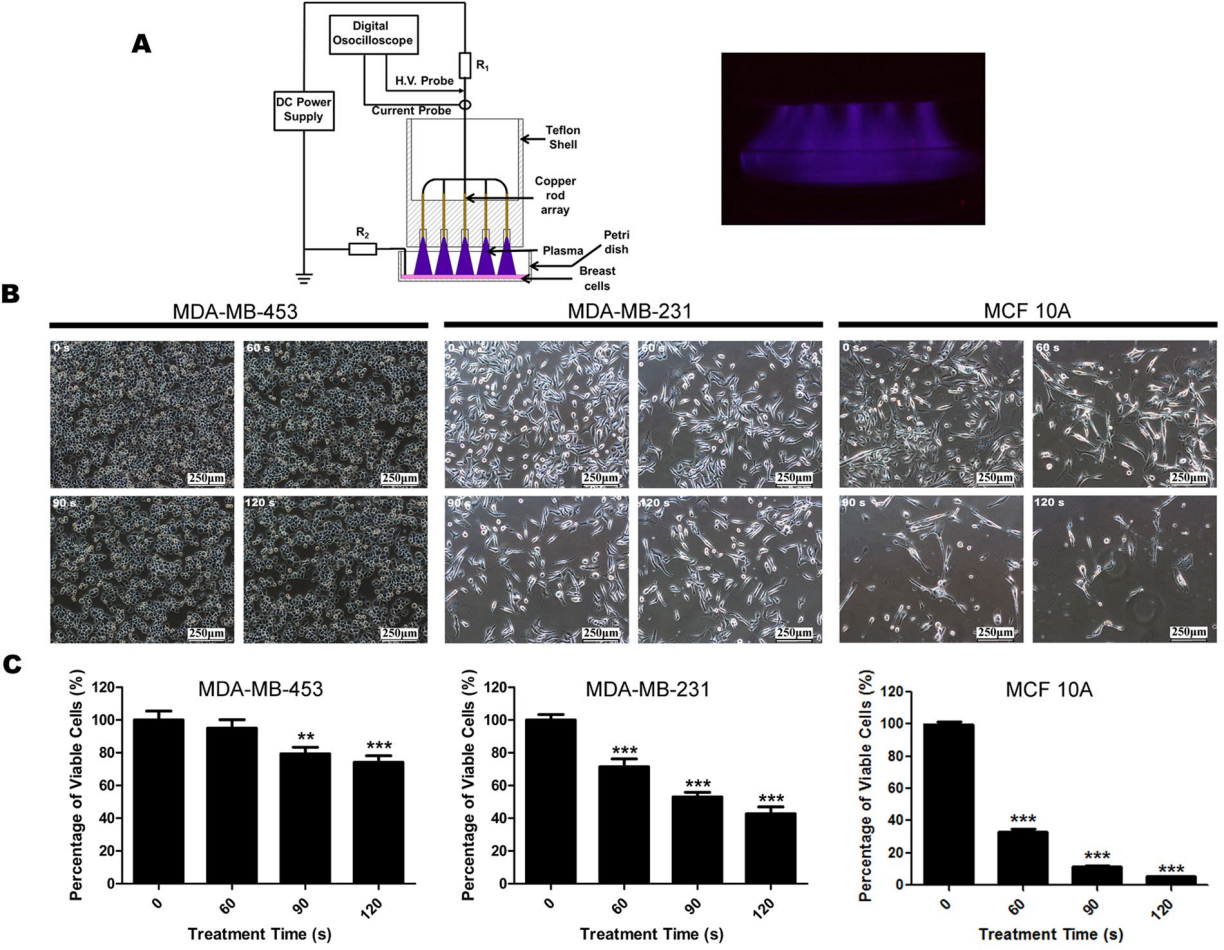
NTP treatment reduces proliferation of MDA-MB-453, MDA-MB-231 and MCF10A cells in a time dose dependent inhibition. (**A**) Schematic of the non-thermal atmospheric plasma set-up utilised in this study, and a photograph of the air plasma. All cells were exposed to NTP for 0, 60, 90 or 120 s. Forty-eight hours later, cell images were captured with 100 × magnification (**B**) and cell numbers of each dish were counted by an automatic analyzer CountStar (**C**). All experiments were replicated a minimum of three times. Data are presented as means ± S.D. and statistical analysis was carried out using one-way ANOVA with Tukey’s multiple comparison test (*p < 0.05, **p < 0.01, ***p < 0.001 versus control).

After 48 hours, following the NTP treatment, the percentage of viable cells was quantified. Both two types of TNBC cell lines MDA-MB-453 and MDA-MB-231 observed a reduction of proliferation (Fig. 1A and B). The results showed that at 60 s NTP treatment MDA-MB-453 had no significant reduction comparing to 0 s treatment (Fig. 1C). Furthermore, at 120 s NTP treatment MDA-MB-453 percentage of viable cells was reduced to <80%, whereas MDA-MB-231 was reduced to <50% (Fig. 1C). In addition, a significant reduction in proliferation was observed in normal TN breast cell lines MCF10A (Fig. 1B). From 60 s to 120 s NTP treatment the percentage of viable cells of MCF10A was all reduced significantly to <40% (Fig. 1C). Morphology of MDA-MB-231 and MCF10A is different from MDA-MB-453 (Fig. 1B), indicating that this difference might be related to the cause of different responses. Previous study has reported that the plasma effects on the cell shape and membrane as well as cell viability points out the complex interaction between plasma and cell membrane surface^42^.

### MDA-MB-453, MDA-MB-231 and MCF10A cells show different response under NTP treatment with or without ROS scavenger NAC

**Figure 2:**
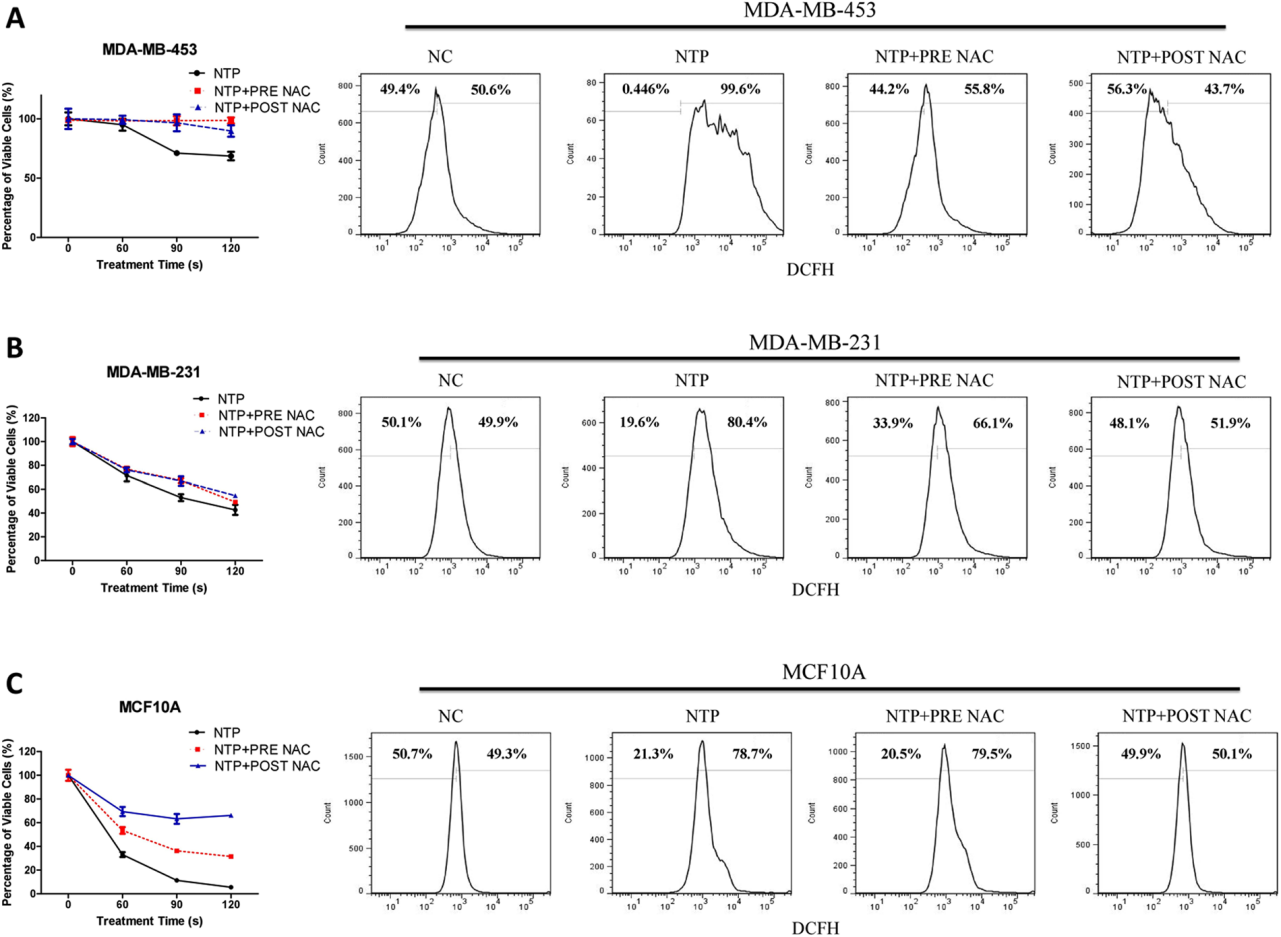
Effects of NTP treatment with or without ROS inhibitor NAC on MDA-MB-453, MDA-MB-231 and MCF10A cells proliferation. Cells were treated with (PRE NAC and POST NAC) or without NAC (NTP) exposed to NTP for 0, 60, 90 or 120 s. Forty-eight hours later, cell numbers of each dish were counted by an automatic analyzer CountStar. ROS fluorescence was analyzed by FACSVerse flow cytometer, 1 h after 120 s NTP treatment. Cells were treated with: Negative Control (NC), 120 s NTP treatment (NTP), pretreat with 5 mM NAC before 120 s NTP treatment (NTP + PRE NAC), post-treat with 5 mM NAC immediately after 120 s NTP treatment (NTP + POST NAC). For improved illustration, a guidance ratio (compared with NC group) has been indicated through histograms. All experiments were replicated a minimum of three times.

According to data shown in Fig. 1, normal cells MCF10A was observed more sensitive to the cytotoxicity induced NTP treatment compared to cancer cells. Because ROS is the main toxicity species induced by NTP, we intend to use ROS scavenger to protect this cytotoxicity effects on MCF10A cells. To determine the role of ROS induced by NTP treatment on TN breast cell lines, all cells were pre-treated for 1 h with 5 mM NAC and then washed before NTP treatment (PRE NAC) as referred to former studies^26, 43^. In addition, we want to use NAC as a long term co-drug for protecting normal cells from ROS toxicity; cells were also post-treated with 5 mM NAC immediately after NTP treatment (POST NAC) until harvested. After further 48 h interesting results were observed, through PRE NAC and POST NAC treatments counteract part of the 90 s NTP killing effects but NTP remaining significant inhibition of MDA-MB-231 cells (Fig. 2B). MDA-MB-453 cells gained a recovery in proliferation under 90 s and 120 s NTP treatments with the help of both PRE NAC and POST NAC treatments (Fig. 2A). On the other hand, PRE NAC and POST NAC treatments can significantly counteract 60 s, 90 s and 120 s NTP killing effects in MCF10A cells (Fig. 2C). It is indicated that normal cells MCF10A show more resistance to the NTP affection especially with POST NAC treatments. We then analyzed the rise of intracellular ROS in all cell lines after 120 s NTP treatment. All cell lines showed a significantly rise of intracellular ROS after 120 s NTP treatment (Fig. 2). With the help of NAC, intracellular ROS of MDA-MB-453 cells remained unchanged compared to control group after NTP treatments (Fig. 2A). However in MCF10A cells, POST NAC group performed more protection compared to PRE-NAC group (Fig. 2C). It is also indicated that ROS plays the key role in the cause of cell death in MCF10A and MDA-MB-453. Interestingly, strong inhibition in MDA-MB-231 cells was observed under any designed NTP treatment in spite of the fact that the rise of intracellular ROS was significantly different between NAC treated group and NTP group (Fig. 2B). POST NAC group of MCF10A cells showed more tolerable to NTP treatment than NTP treatment without NAC (Fig. 2C). On the other hand, NTP anti-cancer effects on TNBC MDA-MB-231 cells were observed uncorrelated with rise of intracellular ROS (Fig. 2B). It is also showed that post-treated NAC group can effectively protect cells form intracellular ROS cytotoxicity and recover the proliferation of MCF10A cells. These findings lead us to focus on the NTP selective effects by using NAC and revealing the mechanism that causes the divergent response between MDA-MB-231 and MCF10A.

### NTP treatment can induce the inhibition of activation of signal transducer and activator of transcription 3 (Stat3) in MDA-MB-231 and MCF10A cells

Constitutive activation of Stat3 protein (tyrosine-phosphorylated Stat3) has been observed in numerous kinds of tumors, including breast cancer. Previous researches have revealed that Stat3 plays an important role in oncogenesis, including enhancement of cell proliferation, inhibition of apoptosis and induction of angiogenesis^44, 45^.

**Figure 3:**
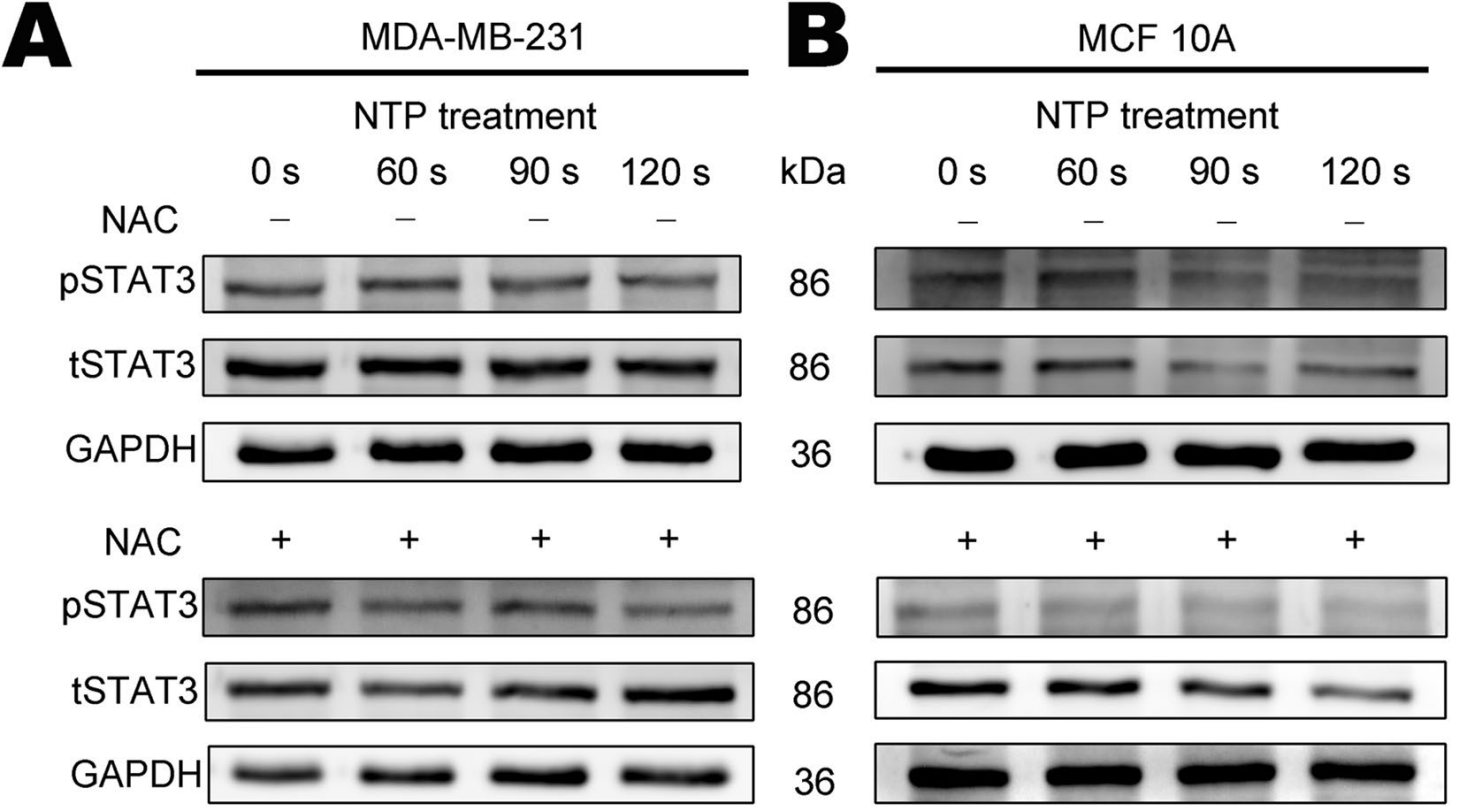
Effects of NTP on the expression of pSTAT3 and tSTAT3 in MDA-MB-231 and MCF10A cells. All cells were exposed to NTP for 0, 60, 90 or 120 s. After forty-eight hours post-treated with 5 mM N-acetyl cysteine (NAC+) or without (NAC−), proteins from total cell lysates were harvested. The expressions of pSTAT3 and STAT3 in MDA-MB-231 (**A**) and MCF10A (**B**) cells were detected by Western blot analysis. GAPDH was taken as a loading control throughout. The gels/blots were processed under the same experimental condition. Cropped gels/blots are displayed and full-length blots/gels are presented in Supplementary Figure 1.

After NTP treatment or post-treatment with ROS scavenger NAC, the expression of tyrosine-phosphorylated Stat3 (pSTAT3) in MDA-MB-231 (Fig. 3A and Supplementary Figure 1A) and MCF10A (Fig. 3B and Supplementary Figure 1B) cells was inhibited. The same mechanism was also confirmed in MDA-MB-453 (Supplementary Figure 2). This indicates that reduction of proliferation in MDA-MB-231 and MCF10A is correlated with the inhibition of activation of Stat3 protein by NTP treatment.

### The ROS affects the proliferation of MDA-MB-231 and MCF10A cells in a different way

**Figure 4:**
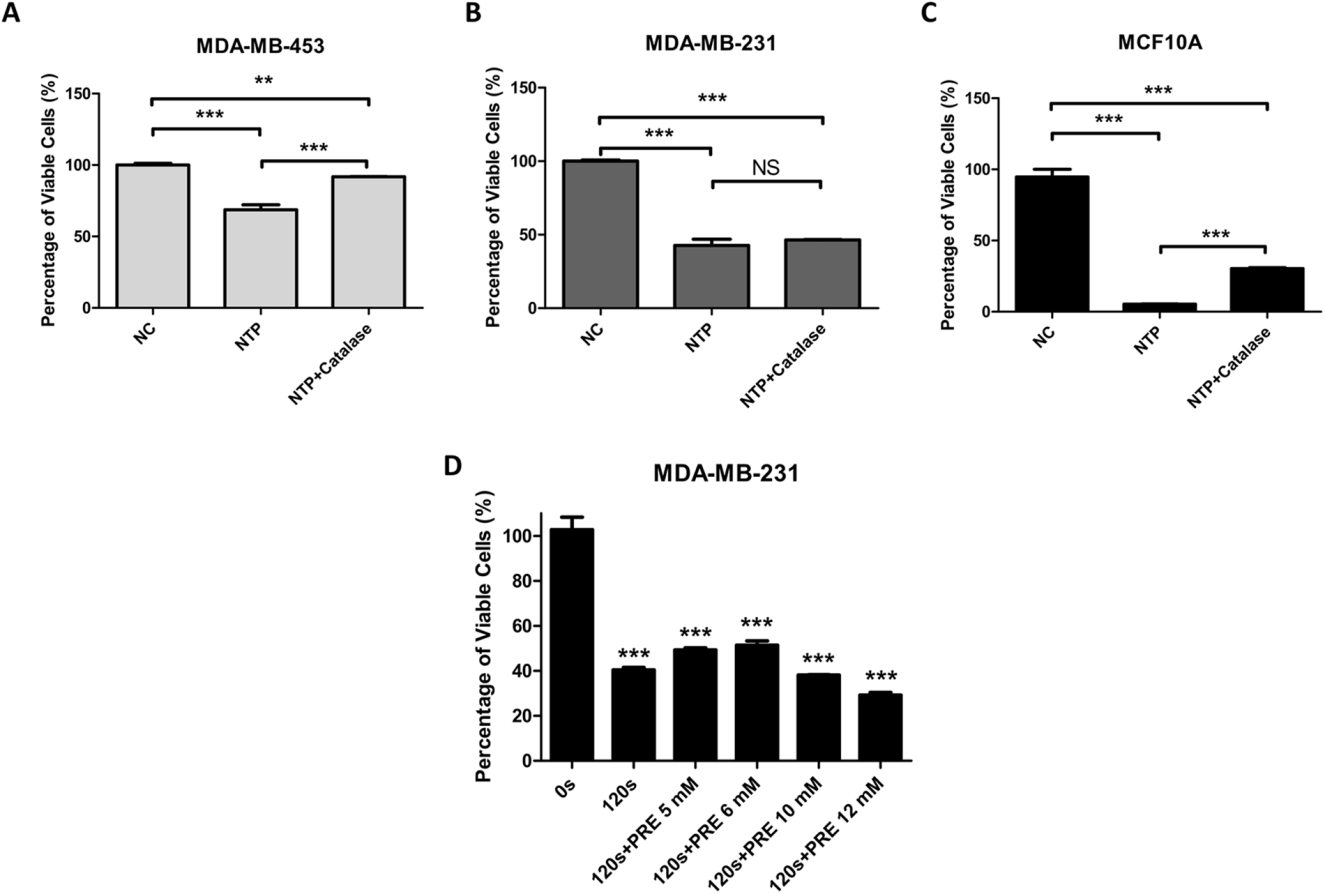
Effects of 120 s NTP treatments with extracellular and intracellular ROS inhibitor on cells’ proliferation. MDA-MB-453 (**A**), MDA-MB-231 (**B**) and MCF10A (**C**) cells were treated: Negative Control (NC), 120 s NTP treatment (NTP) without or with 500 U/ml catalase (NTP + Catalase). MDA-MB-231 cells (**D**) were treated: exposed to NTP for 0 and 120 s, pretreat with 5, 6, 10 and 12 mM NAC before 120 s NTP treatment. After forty-eight hours, cell numbers of each dish were counted by an automatic analyzer CountStar. All experiments were replicated a minimum of three times. Data are presented as means ± S.D. and statistical analysis was carried out using one-way ANOVA with Tukey’s multiple comparison test (NS = No significance, *p < 0.05, **p < 0.01, ***p < 0.001).

To reveal the mechanism that makes MDA-MB-231 and MCF10A behavior differently under NTP treatment and to confirm the role of ROS in NTP treatments, catalase as extracellular ROS scavenger was employed in 120 s NTP treatments firstly. Catalase was adding into medium just before 0 s and 120 s NTP treatments. MDA-MB-453 cells gained resistance to NTP treatment with catalase (Fig. 4A). MCF10A cells got a recovering in proliferation (Fig. 4C). Interestingly, MDA-MB-231 still remained sensitive to NTP treatment (Fig. 4B) and which was similar to PRE NAC and POST NAC treatments (Fig. 2B). On the other hand, MCF10A cells with POST NAC treatment (Fig. 2C) can counteract more NTP killing effects than catalase treatment (Fig. 4C).

According to Fig. 2B results, PRE NAC treatments would counter part of intracellular ROS cytotoxicity by 120 s NTP treatment (from about 42 to 49 percent of viable cells). Furthermore, we pre-treated MDA-MB-231 cells with higher concentration of NAC to confirm that MDA-MB-231 cells are resistant to 120 s NTP treatments’ intracellular ROS cytotoxicity but not killing effects. It was showed that 10 mM and 12 mM NAC were too high to cause cytotoxicity to cells and 6 mM NAC still cannot counteract most part of the killing effect induced by NTP (Fig. 4D). It also observed that MDA-MB-231 cells pre-treated with 6 mM NAC results in about 51 percent of viable cells after 120 s NTP treatment, which is similar to 5 mM NAC results (49 percent of viable cells). Results of Fig. 4B and D showed that NTP anti-cancer capacity beyond ROS cytotoxicity is still effective on MDA-MB-231 cells.

**Figure 5:**
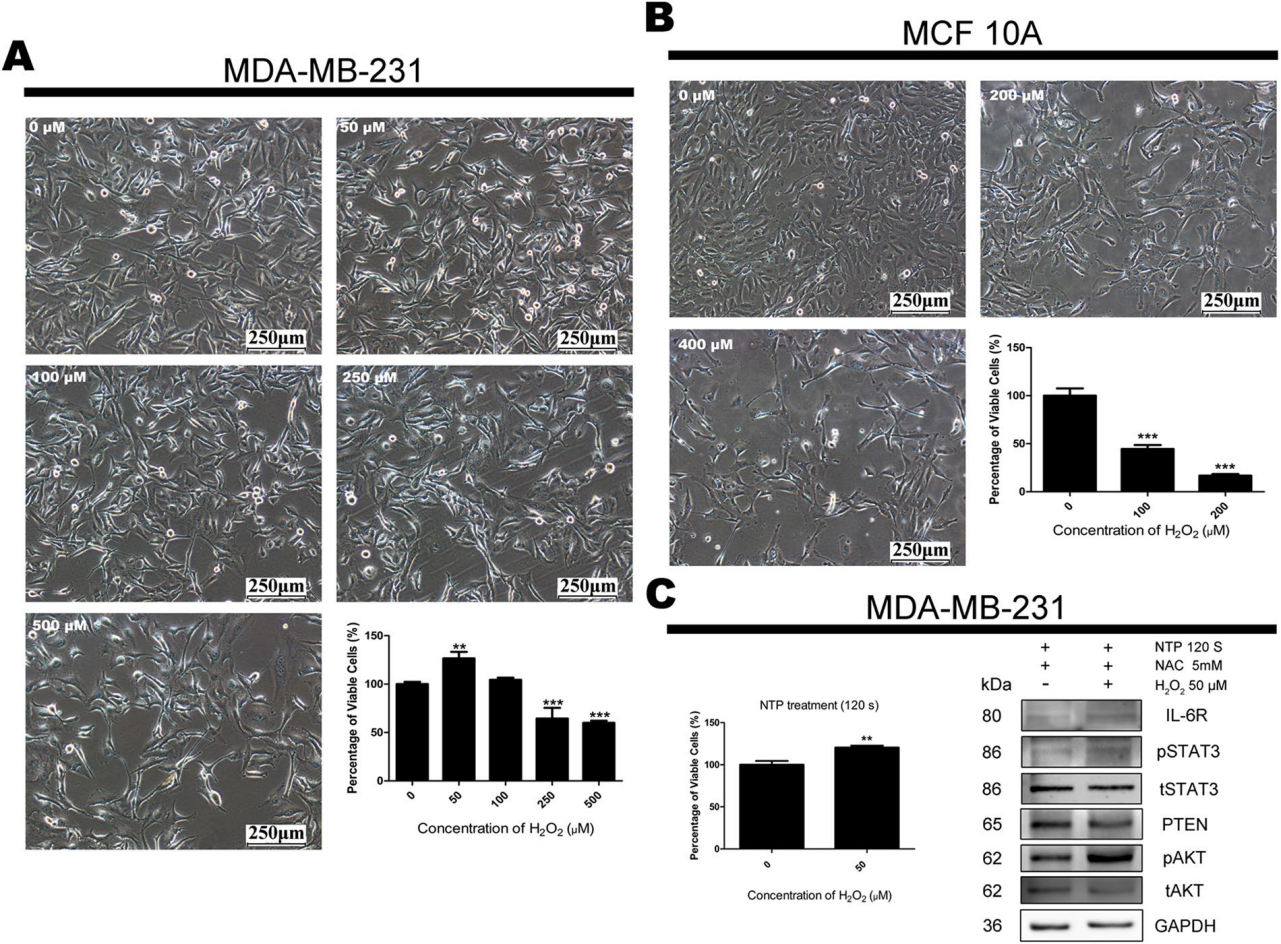
Effects of H_2_O_2_ on the proliferation of MDA-MB-231 and MCF10A cells. MCF10A (**A**) and MDA-MB-231 (**B**) cells were incubated with multi-dose H_2_O_2_. Forty-eight hours later, cell images were captured with 100 × magnification and cell numbers of each dish were counted by an automatic analyzer CountStar. All experiments were replicated a minimum of three times. Data are presented as means ± S.D. and statistical analysis was carried out using one-way ANOVA with Tukey’s multiple comparison test. MDA-MB-231 cells were post-treated with 5 mM NAC and 0/50 μM H_2_O_2_ immediately after 120 s NTP treatment (**C**). Cell numbers of each dish were counted by an automatic analyzer CountStar and proteins from total cell lysates were harvested after forty-eight hours. Data are presented as means ± S.D. for three independent experiments and analyzed by Student’s t-test. *p < 0.05, **p < 0.01, ***p < 0.001 versus control. The expressions of IL-6R, pSTAT3, tSTAT3, PTEN, pAkt and tAkt were detected by Western blot analysis. GAPDH was taken as a loading control throughout. The gels/blots were processed under the same experimental condition. Cropped gels/blots are displayed and full-length blots/gels are presented in Supplementary Figure 4.

Additionally, H_2_O_2_ multi-dose experiments were examined. Under high concentration of H_2_O_2_ treatments, the proliferation of both MDA-MB-231 and MCF10A cells observed a significantly decreasing (Fig. 5A and B). We also examined H_2_O_2_ multi-dose experiments with 5 mM NAC and it was showed that 5 mM NAC could counteract part of 50 μM and 100 μM H_2_O_2_ cytotoxicity but not when the concentration of H_2_O_2_ reaches 150 μM (Supplementary Figure 3A). As we measured the H_2_O_2_ concentration of 120 s NTP treated medium is 185 μM (Supplementary Figure 3B). And it was observed that all three cell lines treated with catalase can counteract 185 μM H_2_O_2_ cytotoxicity. Meanwhile 5 mM NAC could not protect cells from cytotoxicity induced by such high H_2_O_2_ concentration (Supplementary Figure 3C), although 5 mM NAC would consume most H_2_O_2_ in 3 hours and eliminate all H_2_O_2_ in 24 hours (Supplementary Figure 3D). Interestingly in MDA-MB-231 cells, low concentration and high concentration of H_2_O_2_ can affect the proliferation in opposite directions (Fig. 5A). MCF10A cells showed no resistance to H_2_O_2_ treatment (Fig. 5B and Supplementary Figure 3) just similar to the situation under NTP treatment (Fig. 1B).

Taken together, it is indicated that NTP shows selective anticancer effects when employed with 5 mM POST NAC treatments and ROS cytotoxicity plays an important role in NTP treatment to those who are sensitive to ROS. Further more, it is important to find out which signaling pathways are affected by NTP treatments.

### Low concentration of H_2_O_2_ recovers the induction of proliferation in MDA-MB-231 cells under 120 s NTP treatment with post-treated NAC by effects Interleukin-6 receptor (IL-6R)/Stat3 pathway

The IL-6/IL-6 receptor complex performs as upstream regulatory of Stat3 pathway^46^ and has important roles in the proliferation of cancer cells^47^. Both of the IL-6 and IL-6R are expressed in MDA-MB-231^48^. Depending upon the results shown in Fig. 5A, MDA-MB-231 cells were post-treated with 5 mM NAC and 0/50 μM H_2_O_2_ immediately after 120 s NTP treatment. The NTP killing effects on both treated groups were still significantly (data not shown), but a significant recovery of proliferation and IL-6R pathway upregulation in low concentration of H_2_O_2_ treated cells was observed (Fig. 5C and Supplementary Figure 4). It indicated that low concentration of H_2_O_2_ can recover the expression of IL-6R and tyrosine-phosphorylated Stat3. Under this situation, cell proliferation related PTEN/Akt pathway was also biased through IL-6R/pSTAT3 affection (Fig. 5C and Supplementary Figure 4). Taken together, the IL-6R expression plays an important role in proliferation of MDA-MB-231 cells and NTP can down regulate IL-6R pathway.

### The mechanism of NTP selective effects on TN normal breast cells MCF10A and TNBC cells MDA-MB-231 with POST NAC treatment

**Figure 6:**
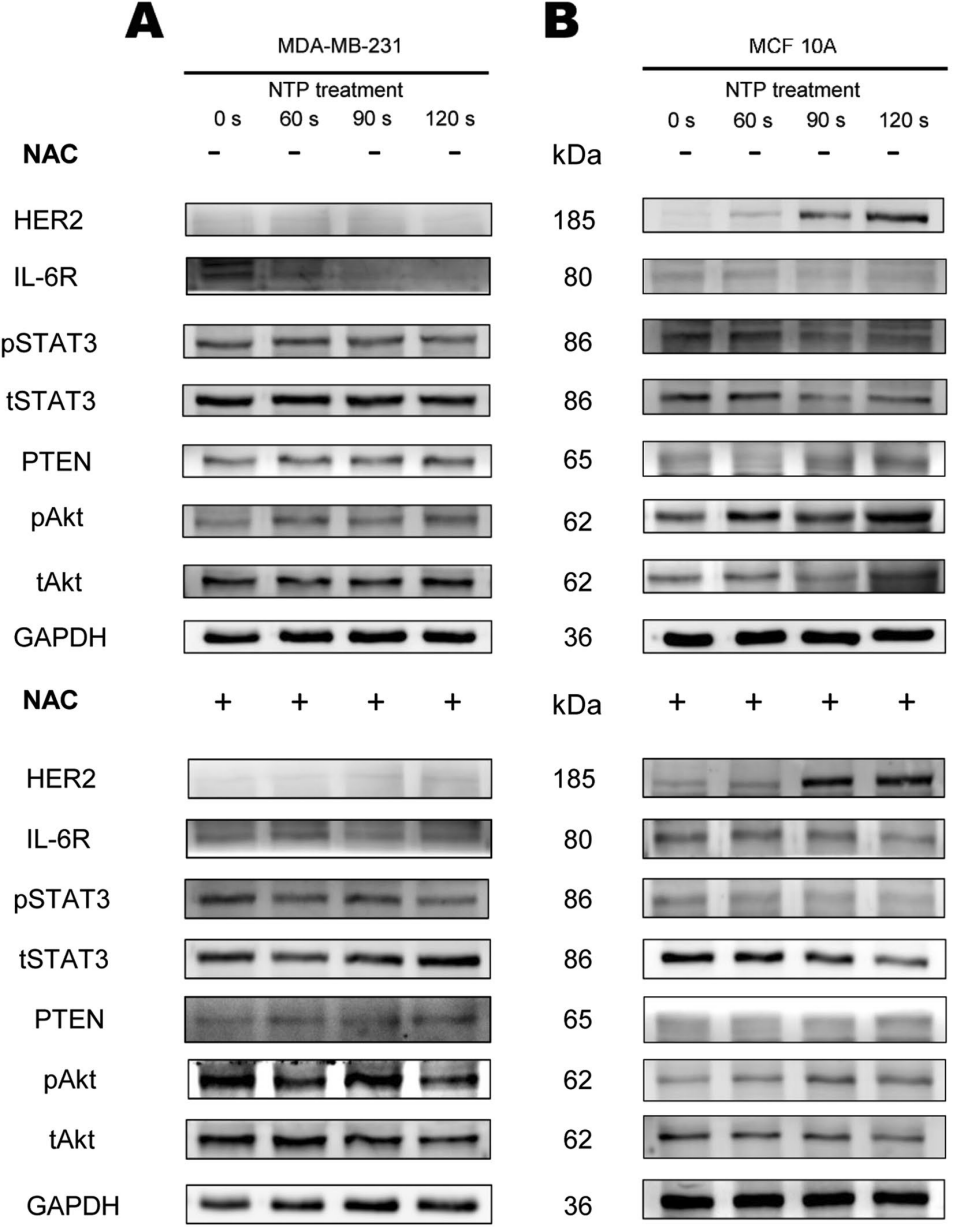
Effects of NTP on the IL-6R/pSTAT3 and HER2/pAkt pathway in MDA-MB-231 and MCF10A cells. MDA-MB-231 (**A**) and MCF10A (**B**) cells were exposed to NTP for 0, 60, 90 or 120 s. After forty-eight hours post-treated with 5 mM N-acetyl cysteine (NAC+) or without (NAC−), proteins from total cell lysates were harvested. The expressions of HER2, L-6R, pSTAT3, tSTAT3, PTEN, pAkt and tAkt were detected by Western blot analysis. GAPDH was taken as a loading control throughout. The gels/blots were processed under the same experimental condition. Cropped gels/blots are displayed and full-length blots/gels are presented in Supplementary Figure 5.

48 h after NTP treatment post-treated with or without NAC, we examined the activation of IL-6R/STAT3 and PTEN/Akt pathways in MDA-MB-231 and MCF10A cells. Along with growing exposure time dose under NTP treatment, the expression of IL-6R and pSTAT3 showed significant decrease while in contrary PTEN increased markedly due to the decline of the former in all cases (Fig. 6A and B). Since NAC eliminated the protection of ROS on Akt activation, the expression of pAkt in MDA-MB-231 is down regulated by NTP induced PTEN increasing (Fig. 6A and Supplementary Figure 5A). It is interesting that Akt activation constantly increased in MCF10A cells which was induced by NTP with POST NAC treatment (Fig. 6B and Supplementary Figure 5B). Previous studies revealed that Akt activation promotes proliferation in MCF10A cells^49^ and HER2 is an upstream regulator of Akt activation^50, 51^. Surprisingly, the expression of HER2 recovered and increased significantly in this TN normal cell MCF10A (Fig. 6B and Supplementary Figure 5B) after NTP treatment with or without NAC. Under the same situation, TNBC cells MDA-MB-231 remain lack of HER2 expression (Fig. 6A and Supplementary Figure 5A). These results suggest that selective effects of NTP treatments on HER2 expression caused the divergent response in MDA-MB-231 and MCF10A after post-treated with NAC.

## Discussion

In recent years, NTP has been demonstrated to be a potential tool in anticancer therapy. The mechanism of NTP effects on cancer cells *in vitro* has been mainly revealed in several aspects, including the toxicity induced by chemical essence of NTP, NTP affects the change of cellular shape and membrane, the transmembrane diffusion of NTP generated reactive species, the change of intracellular ROS and redox balance, causing DNA damage and influencing apoptosis pathways^52^. Previous studies also reported that NTP treatment can induce apoptotic cell death in various tumors by ROS generation intracellular and extracellular. Mechanism of these ROS-dependent cell deaths by NTP was discovered to have been associated with several cellular signaling pathways such as: NTP treatment induced p53-dependent apoptosis in colon carcinoma cells^53, 54^. PARP1-mediated DNA damage is also an extensively revealed mechanism that explains the mammalian and carcinoma cell death induced by non-thermal plasma^37, 55^. Additionally, several groups have reported that activation of ERK and JNK signaling pathway is involved in immune cells, lung and cervical cancer cells apoptosis by non-thermal plasma^15, 24, 56^. Taken together, these studies reveal that the NTP associated mechanisms is complicated and specific in different types of cells. It is notable that to reveal mechanisms specifically in such subgroups of cancer under non-thermal plasma treatment is critical for further precision therapy.

**Figure 7:**
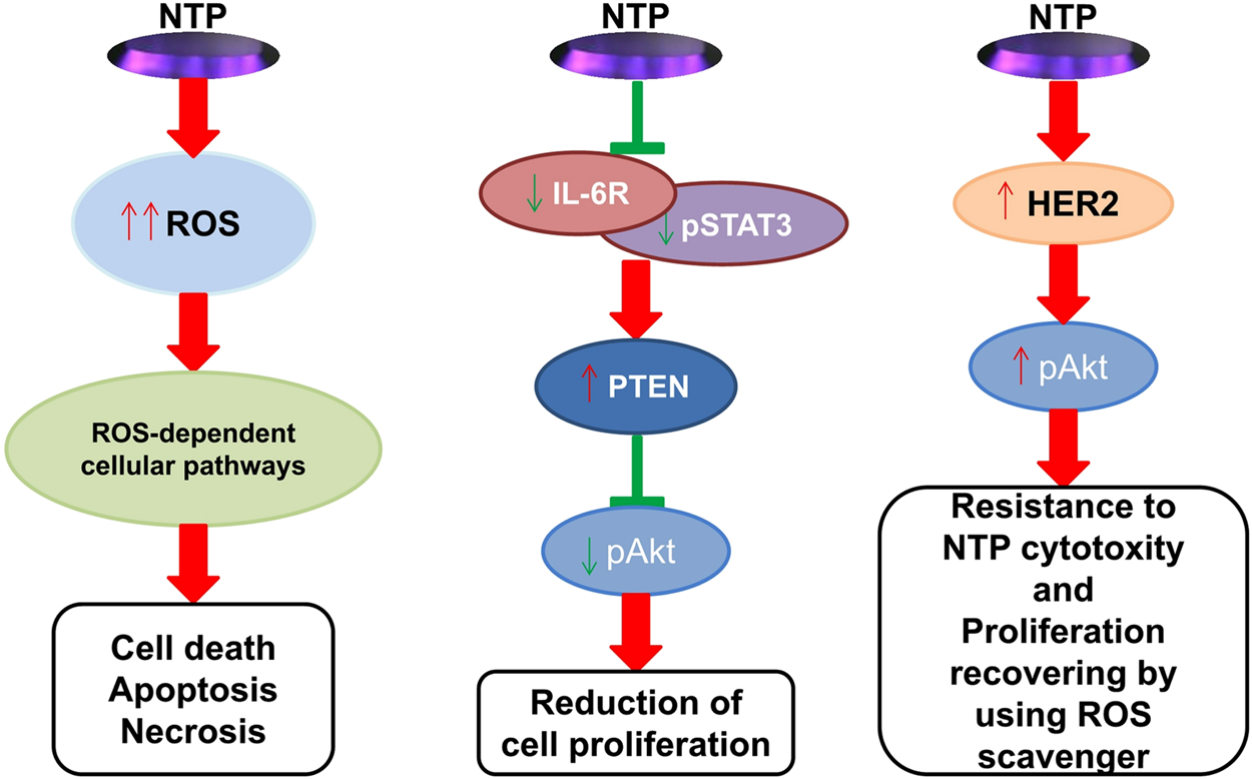
A general survey of NTP treatment affection on triple-negative breast carcinoma and normal cells through different cellular pathways.(**A**) NTP treatment inhibits proliferation of MDA-MB-453 and MCF10A cells through ROS-dependent cellular pathway (not discussed in this article). (**B**) NTP treatment effects on reduction of proliferation through IL-6R/pSTAT3 pathway in MDA-MB-231 and MCF10A cells. (**C**) NTP treatment effects on recovering the proliferation especially in normal breast MCF10A cells with post-treated 5 mM NAC.

Patients with TNBC, a subgroup of breast cancer, lack specific therapy and have a comparatively poor outcome^57^. Especially subtypes of TNBC like MDA-MB-231 cells are more invasive and metastasize because of constantly activated IL-6 signaling^58^. IL-6 receptor plays a critical role in IL-6 signaling in human mammary carcinoma cell lines^48^. Depending on our results, NTP treatment can significantly inhibit IL-6R/pSTAT3 activation *in vitro* (Fig. 7B). Based on this conclusion, it is highly possible that NTP could be a promising therapy for cancer related with IL-6 signaling.

Activated Akt gets involved in the regulation of cellular growth and cell survival. In cancer cells, Akt activation has been associated with increased resistance to apoptosis. On the other hand, Akt can be activated by some antitumor cytokine^59^. Recent study reported that pAkt in thyroid papillary cancer cells can be decreased by NTP treatment^60^. We also confirmed that Akt activation can be reduced after NTP treatment with NAC (Fig. 7B). In addition, we observed that Akt can be constantly activated especially in normal breast MCF10A cells through NTP effects (Fig. 7C). We discover this selective effect of NTP on Akt inactivation and activation was observed.

Non-thermal atmospheric plasma treatment is widely reported to cause cytotoxic effects in cells by inducing RONS to the cell culture^43, 61, 62^. Our data showed that the inhibition of proliferation in normal breast cell MCF10A cells induced by 120 s NTP treatment was similar to the cytotoxicity of 200 μM H_2_O_2_ treatment. In order to avoid ROS cytotoxicity on normal cells MCF10A induced by NTP, we have employed 5 mM NAC post-treated with cells after NTP treatments that could recover MCF10A’s cell viability from about 5% to 65% (120 s NTP) and a significant prevention of intracellular ROS (Fig. 2C). Results of Fig. 2C also showed that POST NAC treatments would recover more proliferation of MCF10A cells than PRE NAC treatments. It was reported that NAC as a derivative of cysteine would strongly react with ROS^41, 63, 64^. Our results showed that POST NAC treatments could protect more MCF10A cells than extracellular ROS scavenger catalase under 120 s NTP treatments (Figs 2C and 4C), but POST NAC treatments could not save cells after 185 μM H_2_O_2_ treatments (Supplementary Figure 3C). In Supplementary Figure 3A, it was observed that post-treated MCF10A cells with 5 mM NAC could counteract part of the cytotoxicity induced by 50 and 100 μM H_2_O_2_ but not 150 μM H_2_O_2_. It may be the case that high concentration H_2_O_2_ (more than 100 μM) could cause permanent damage in a short period of time before NAC consume it. Taken together, these findings indicated that HER2/Akt activation induced by NTP treatments which plays a crucial role in proliferation recovery of MCF10A cells. Interestingly, TNBC MDA-MB-231 cells show different responses to H_2_O_2_ under low and high concentrations. The similar results are observed in *C. elegans* ^65^ and Hepatic cells mice^66^ through different cell signaling. Our findings showed that low concentration of H_2_O_2_ can counteract part of NTP killing effects on TNBC MDA-MB-231 cells via IL-6R/pSTAT3 pathway. Based on these findings above, generation of ROS accompany with NTP treatment may have an influence of unexpected to some TNBC patients.

To comprehend the mechanism underlying the selective effects of non-thermal atmospheric plasma are essential in utilizing it to clinical TNBC therapy. In conclusion, our experiments have demonstrated here that the proliferation of TNBC cells can be significantly reduced after NTP treatment in two different ways. TNBC like MDA-MB-453 cells, who are lacking of autocrine IL-6, can be affected through ROS cytotoxicity induced by NTP treatment (Fig. 7A). TNBC like MDA-MB-231 cells, who are not sensitive to ROS and combined with high expression autocrine IL-6, can be inhibited through IL-6R/pSTAT3 pathway under NTP treatment (Fig. 7B). With the help of ROS scavengers, constantly HER2/Akt activation in normal TN cells like MCF10A making cells survive through selective effects of NTP treatment (Fig. 7C). These surprising fundamental results suggest that the selective effects by restricted NTP treatment can be further developed into a novel approach for urgently needed TNBC therapies.

Our results indicate that NTP remains significant in inhibition of TNBC MDA-MB-231 cells even with the ROS blockader NAC. In addition, we reported that the mechanism of NTP anti-cancer selectivity relates to constantly HER2/Akt activation induced by NTP especially in MCF10A cells but not in MDA-MB-231 cells. This aspect of NTP effects makes it possible to become a novel tool in non-invasive anticancer therapy. The mechanism of how NTP treatment affects the tumor is still not fully understood. Our experiments demonstrate ROS generation of NTP treatment is important in TNBC subtype like MDA-MB-453 cells therapy, but ROS scavenger employed in NTP treatments should be considered much more important in TNBC subtype like MDA-MB-231 cells therapy. Thus precision therapy for various carcinoma subtypes should be developing further in NTP treatment.

## Methods

### Cell culture

Human breast cancer cell lines MDA-MB-453, MDA-MB-231 and mammalian breast epithelial cell line MCF10A were purchased from the American Type Culture Collection (ATCC). MDA-MB-453 and MDA-MB-231 cells were cultured in L-15 medium supplemented with 10% fetal bovine serum at 37 °C incubator. MCF10A cells were cultured in high glucose DMEM/F12 50:50 mixture supplemented with 5% horse serum, Epidermal Growth Factor (100 mg/ml), Hydrocortisone (1 mg/ml), Cholera Toxin (1 mg/ml) and Insulin (10 mg/ml) at 37 °C and 5% CO_2_ incubator. N-Acetyl L-cysteine (NAC) and catalase was used as ROS scavenger. NAC cytotoxicity to all cell lines was tested and 5 mM NAC was chosen to do further experiments (Supplementary Figure 6A). Catalase cytotoxicity to all cell lines was tested and 500 U/ml catalase was chosen to do further experiments (Supplementary Figure 6B).

### NTP device and treatment

The air plasma is generated between the high voltage electrode (an array with 16 fine copper rods) and culture medium which is used as the ground electrode. The high voltage electrode is driven by 10 kV and the discharge current is about 5 mA being restricted by two ballast resistors R1 and R2 (both 25 mΩ). The gas temperature of the plasma is below 300 K because of its low power consumption. Thus the air plasma could be touched safely and directly by human skin. (Fig. 1A).

4 × 10^5^ cells were seeded into 60 mm diameter petri dishes for plasma exposures. The medium was replaced with 5 ml new complete culture medium just before exposures. The distance between the copper rods and the surface of the medium was fixed at 20 mm. The cells were then exposed to NTP for 0, 60, 90 or 120 s. After 48 hours, the cells were harvested for further experiments.

### Cell counting

Cell viability was determined by trypan blue staining. Cell numbers of each dish were counted by an automatic analyzer (Countstar, China).

### ROS scavenger studies

Cells were pre-treated for 1 h with 5 mM NAC and will be washed before the NTP treatment (PRE NAC), or post-treated with 5 mM NAC immediately after NTP treatment (POST NAC) until harvested for further experiments. As indicated in Fig. 2.

### Detection of intracellular ROS

Intracellular ROS was determined using an oxidation-sensitive fluorescent probe (DCFH-DA) (Beyotime, China). Cells were preloaded with 10 μM DCFH-DA for 30 min and will be washed before NTP treatment. One hour after treatment, cells were harvested and fluorescence was analyzed by FACSVerse flow cytometer (Becton Dickinson, USA). For each sample 30 000 events were collected.

### Western blot analysis

4 × 10^5^ cells of all TN breast cell lines were seeded into 60 mm dishes with 5 mL complete culture medium and then exposed to NTP for 0, 60, 90 and 120 s with or without 5 mM NAC, and 120 s NTP treatment with or without 50 μM H_2_O_2_. After 48 h incubation, the total protein was extracted by modified RIPA lysis buffer (pH 7.4, 1% NP-40, 0.25% Na-deoxycholate, 1 mM EDTA, 150 mM NaCl, 50 mM Tris, protease inhibitor cocktail (Roche)). Proteins from total cell lysates were uploaded and separated by 10% SDS-PAGE, and then electro-transferred to polyvinylidenedifluouride (PVDF) membrane (Millipore Corp, Atlanta, GA, US). The membranes were blocked in 5% non-fat dry milk in PBS/Tween-20 at room temperature for 1 h, and then blotted with primary antibodies overnight at 4 °C, followed by HRP-conjugated secondary antibodies at room temperature for 1 h. The signals were developed using the ECL (enhanced chemiluminescence) reagents (Pierce, Rockford, IL, USA) and read on ImageQuant LAS4000 (GE Healthy).

### Statistical analysis

All experiments were repeated at least three times and all data were presented as mean ± SD (standard deviation). The difference between two groups was analyzed by Student’s t-test, and the difference between more than two groups was analyzed using one-way ANOVA test with Tukey’s multiple comparison test. Any case *p* < 0.05 was regarded as significant.

## Electronic supplementary material

Supplementary Information
