# Supplementary material for: Selective effects of non-thermal atmospheric plasma on triple-negative breast normal and carcinoma cells through different cell signaling pathways
Source: Sci Rep. 2017 Aug 11;7:7980. doi: 10.1038/s41598-017-08792-3 (PMC5554176; doi:10.1038/s41598-017-08792-3)
Supplement: Supplementary file 1 — Supplementary Information [file 41598_2017_8792_MOESM1_ESM.pdf]

Supplemental Material

Selective effects of non-thermal atmospheric plasma on triple-negative breast normal and carcinoma cells through different cell signaling pathways

Yuan Liu[1], Sheng Tan[2, 3], Hao Zhang[1], Xiangjun Kong[2, 3], Lili Ding[1], Jie Shen[4], Yan Lan[4], Cheng Cheng[4*], Tao Zhu[2, 3*] and Weidong Xia[1*]

[1]School of Life Science, University of Science and Technology of China, Hefei, Anhui 230027, China

[2]The CAS Key Laboratory of Innate Immunity and Chronic Disease, School of Life Sciences, University of Science and Technology of China, Hefei, Anhui 230027, China

[3]Hefei National Laboratory for Physical Sciences at Microscale, Hefei, Anhui 230027, China

[4] Institute of Plasma Physics, Chinese Academy of Sciences, P. O. Box 1126, Hefei 230031, P. R. China

E-mail: chengcheng@ipp.ac.cn (C Cheng); zhut@ustc.edu.cn (T Zhu); xiawd@ustc.edu.cn (WD Xia)

Figure S1. Full-length gels\blots of Figure 3.

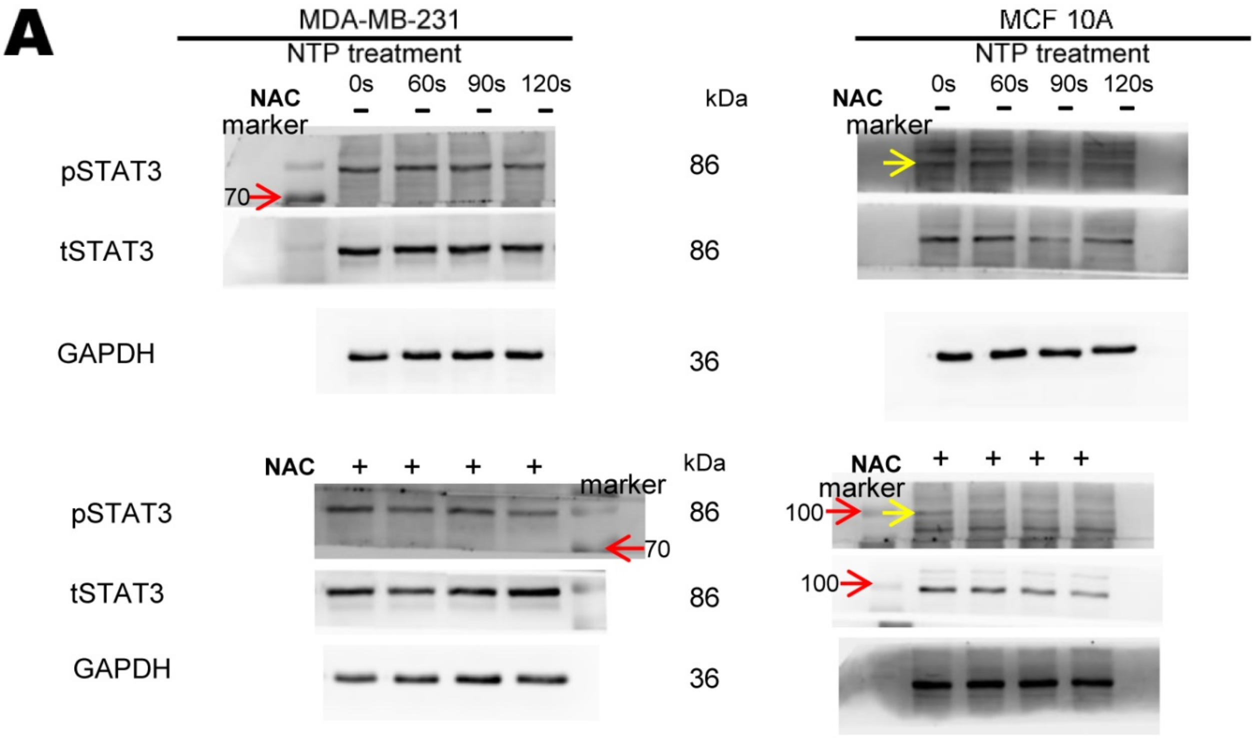

Figure S2. Effects of NTP on the expression of pSTAT3 and tSTAT3 in MDA-MB-453 cells.

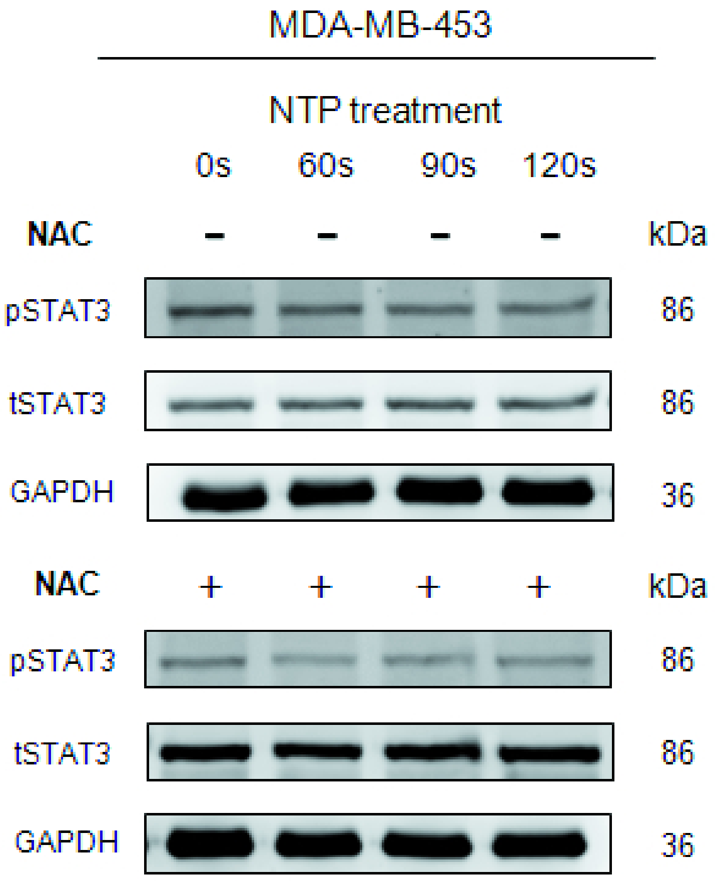

All cells were exposed to NTP for 0, 60, 90 or 120 s. After forty-eight hours post-treated with 5 mM N-acetyl cysteine (NAC+) or without (NAC-), proteins from total cell lysates were harvested. The expressions of pSTAT3 and STAT3 in MDA-MB-453 cells were detected by Western blot analysis. GAPDH was taken as a loading control throughout.

Figure S3. H_2O_2 effects on cells with or without ROS scavenger.

A

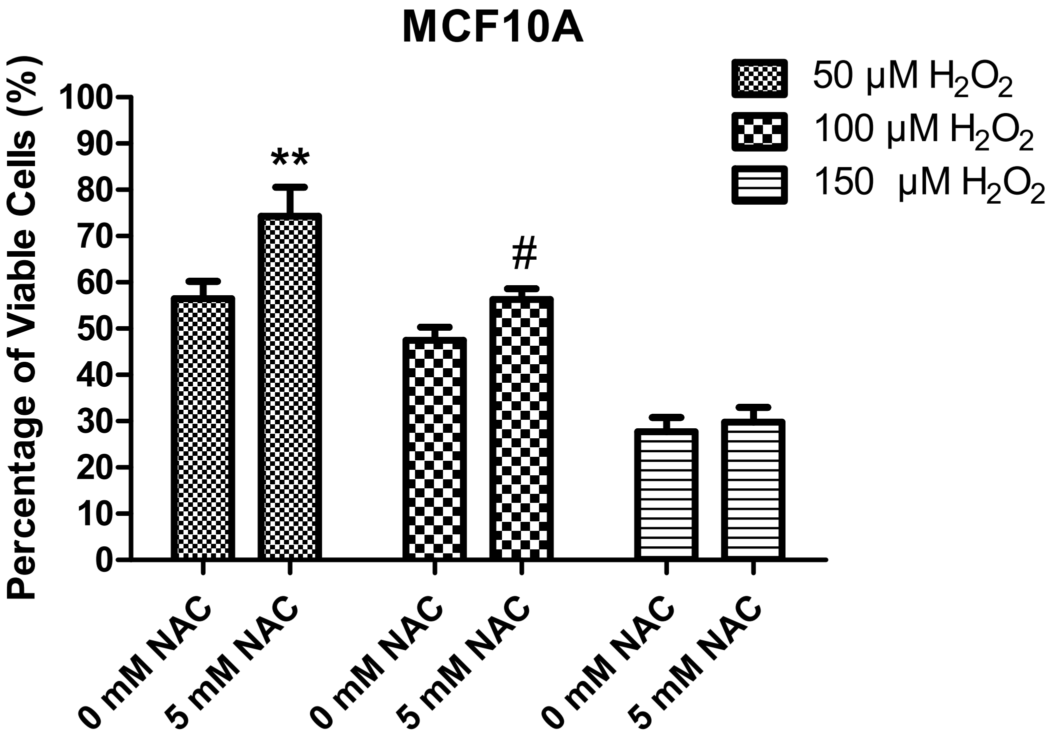

A. MCF10A cells were treated with : 50 μM H_2O_2, 100 μM H_2O_2, 150 μM H_2O_2, and with 0 mM or 5 mM NAC. After forty-eight hours, cell numbers of each dish were counted by an automatic analyzer CountStar. All experiments were replicated a minimum of three times. Data are presented as means ± S.D. and statistical analysis was carried out using one-way ANOVA with Tukey's multiple comparison test (*$p<0.05$, **$p< 0.01$， #$p< 0.05$ versus control).

B

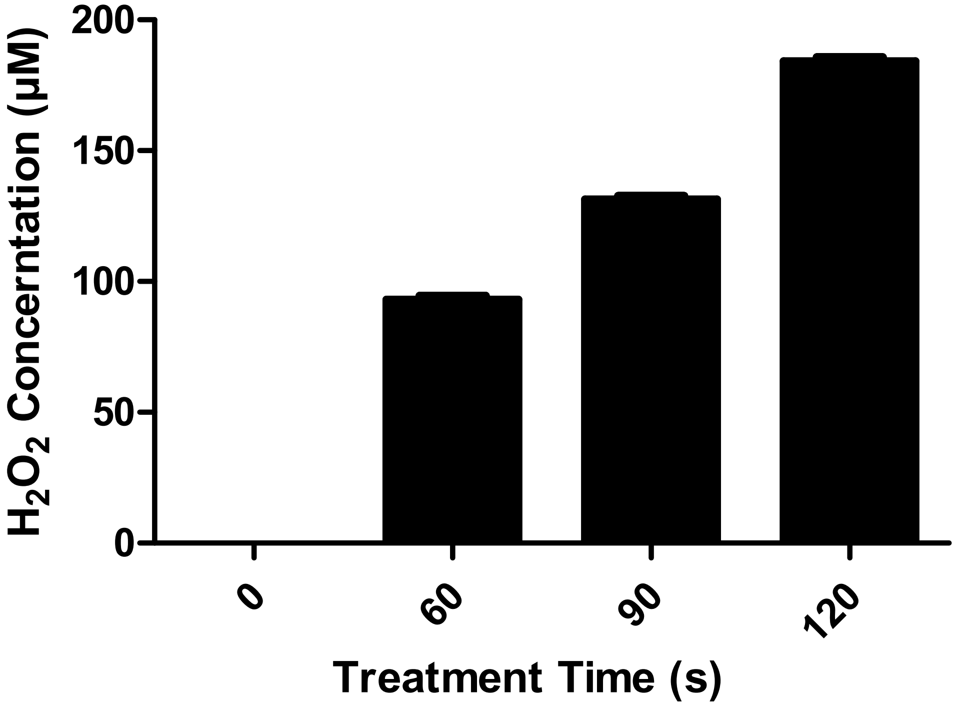

B. H_2O_2 concentration of the NTP treated medium. The H_2O_2 concentration of the NTP treated medium was measured after 60 s, 90 s and 120 s NTP treatment (The test kits was 18789 respectively and the test methods were according to the manufacturer's instructions).

C

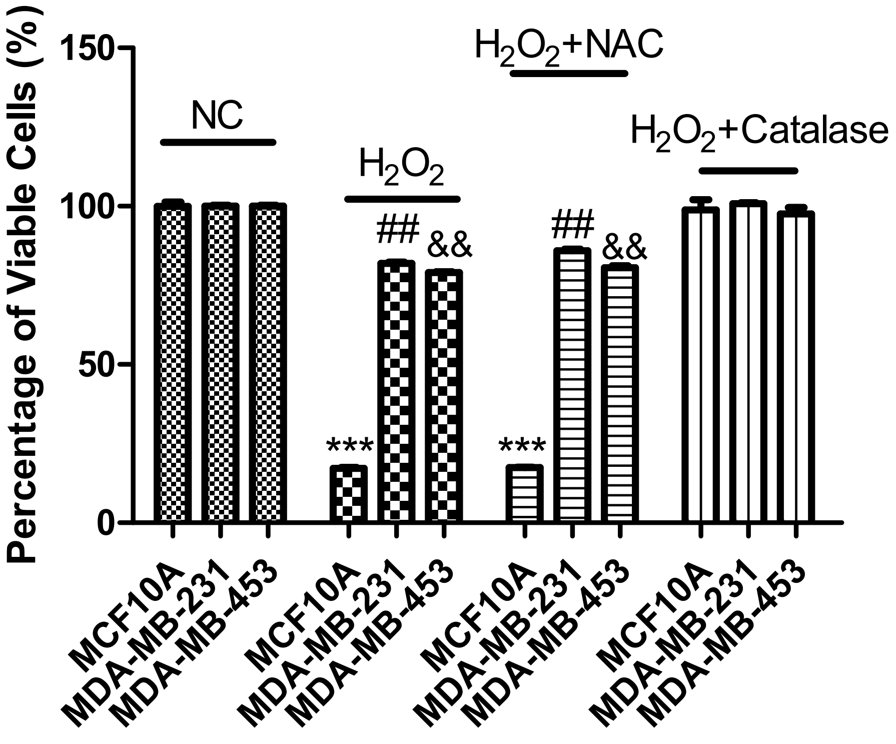

C. All cells were treated with : Negative control (NC), 185 μM H_2O_2 (H_2O_2), 185 μM H_2O_2 and 5mM NAC (H_2O_2+NAC), 185 μM H_2O_2 and 500 U/ml catalase (H_2O_2+Catalase). After forty-eight hours, cell numbers of each dish were counted by an automatic analyzer CountStar. All experiments were replicated a minimum of three times. Data are presented as means ± S.D. and statistical analysis was carried out using one-way ANOVA with Tukey's multiple comparison test (*$p<0.05$, **$p< 0.01$, ***$p< 0.001$，##$p< 0.01$, &&$p< 0.01$, versus control).

D

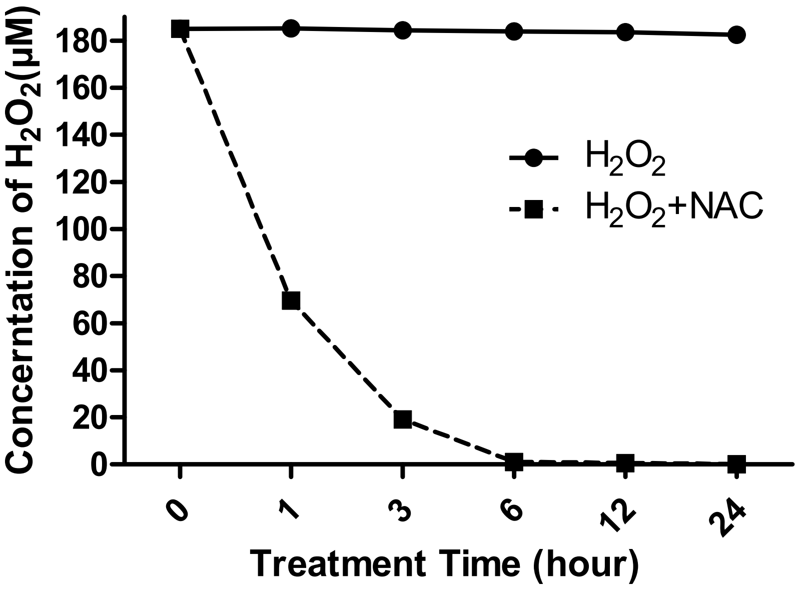

D. The concentration change of 185 μM H_2O_2 with 5 mM NAC.185μM H_2O_2 and 185μM H_2O_2 with 5 mM NAC were dissolved in 1×PBS and incubated at 37 °C incubator.The concentration of H_2O_2 was measured at 0 h, 1 h, 3 h, 6 h, 12 h and 24 h by H_2O_2 Quantitative Assay Kit (Sangon Biotech, China). Data are presented as means ± S.D. for three independent experiments.

Figure S4. Full-length gels\blots of Figure 5C.

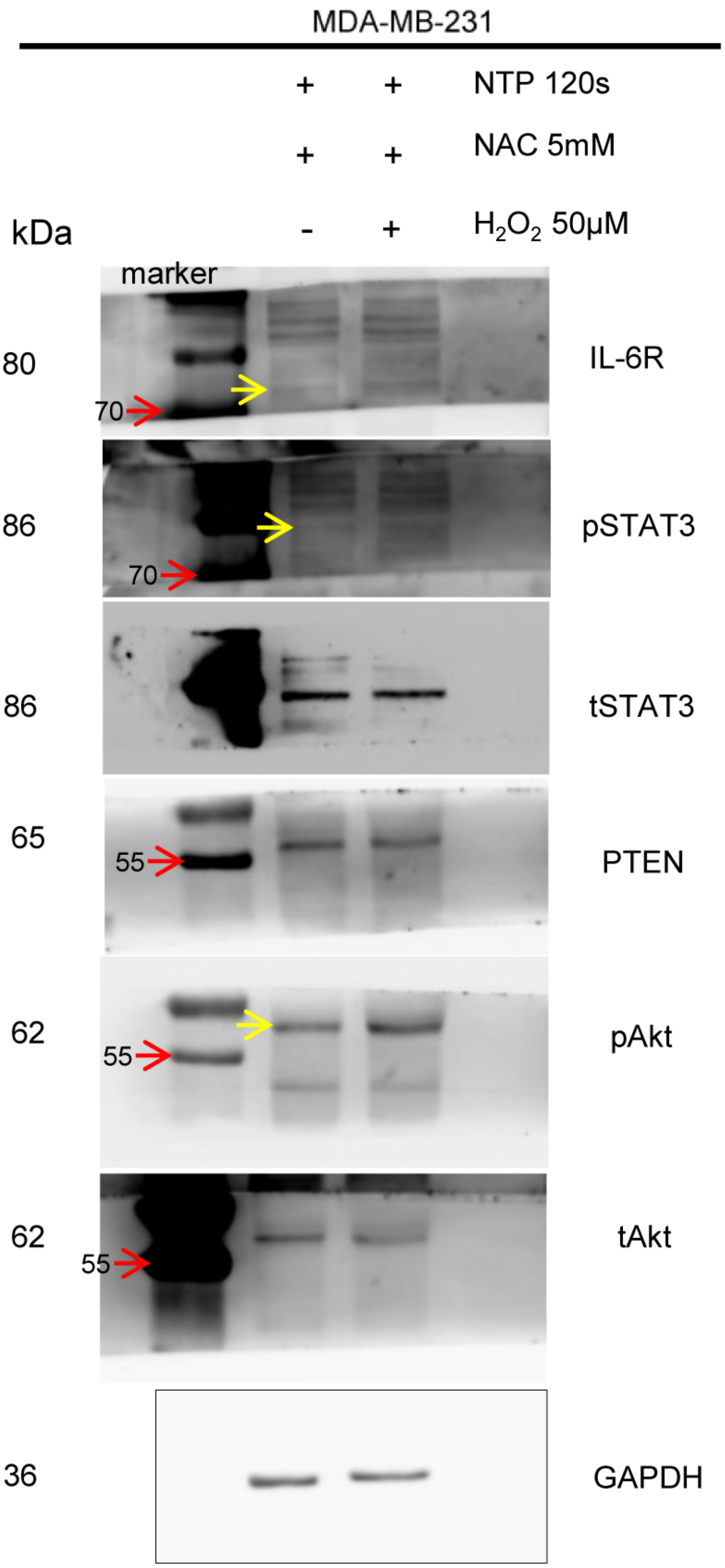

Figure S5. Full-length gels\blots of Figure 6.

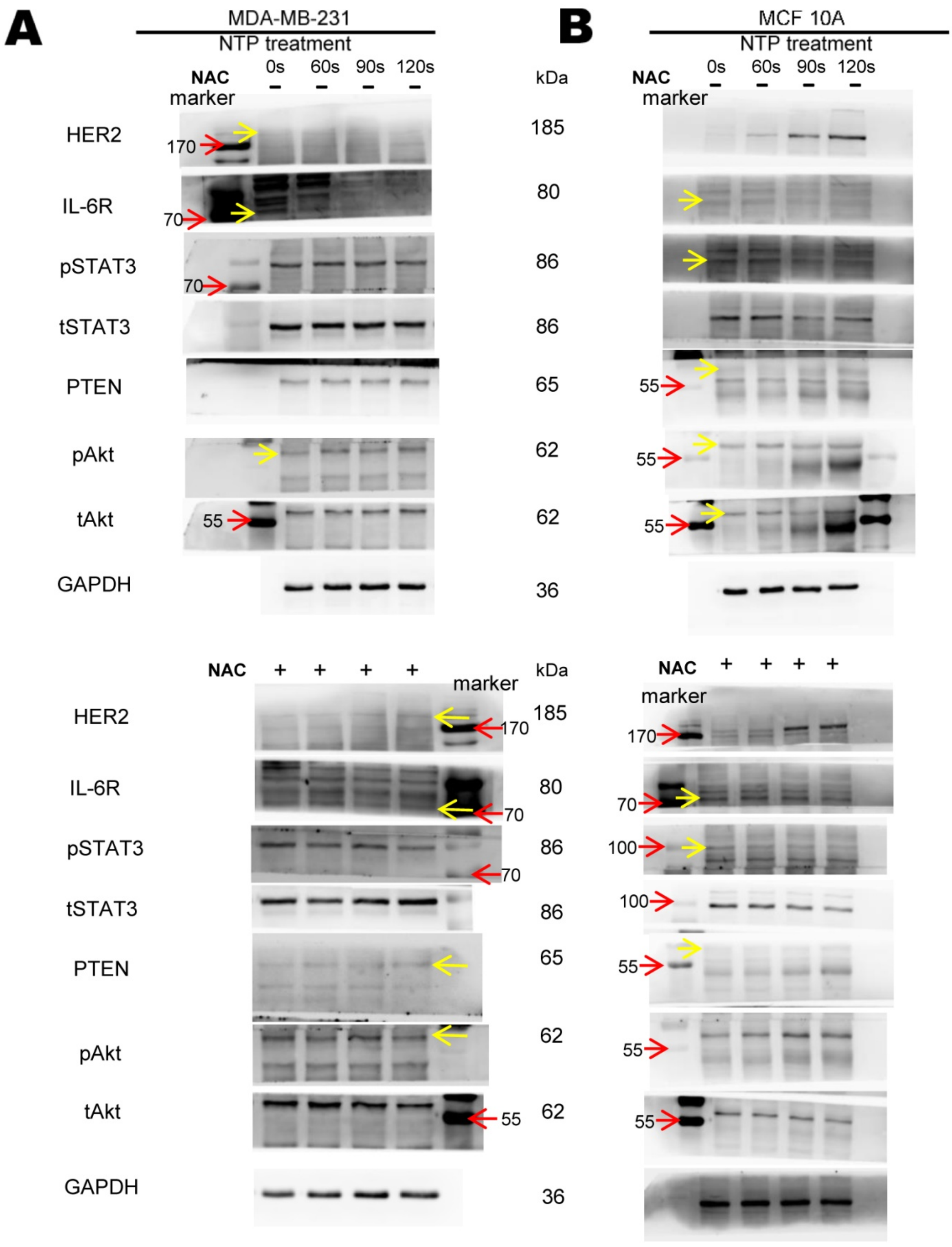

Figure S6. ROS scavenger cytotoxicity test.

A

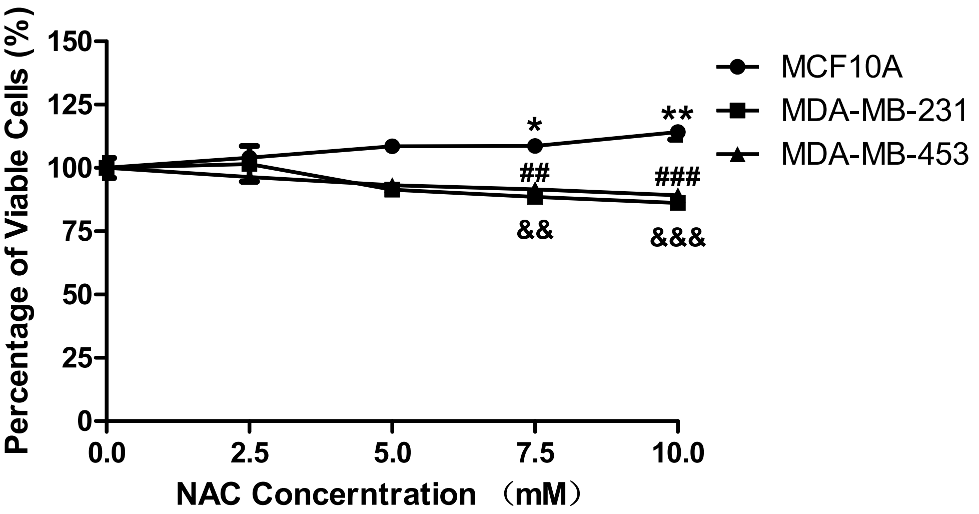

A. All cells were treated with 0mM, 2.5 mM, 5 mM, 7.5 mM, 10 mM NAC. After forty-eight hours, cell numbers of each dish were counted by an automatic analyzer CountStar. All experiments were replicated a minimum of three times. Data are presented as means ± S.D. and statistical analysis was carried out using one-way ANOVA with Tukey's multiple comparison test (*p<0.05, **p< 0.01, ***p< 0.001, ##p< 0.01, ###p< 0.001, &&p< 0.01, &&&p< 0.001 versus control). Each data were compared with relevant 0mM NAC treatment group data.

B

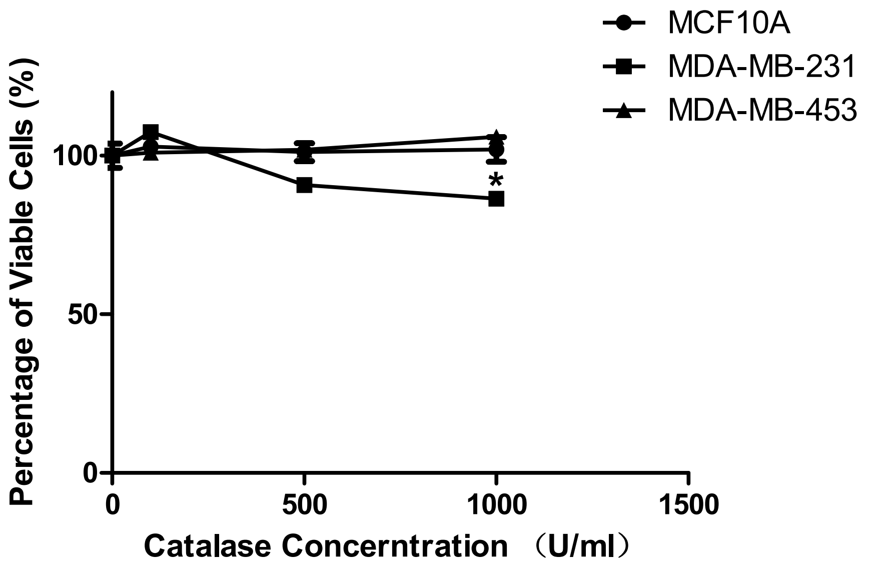

B. All cells were treated with 0 U/ml, 100 U/ml, 500 U/ml, 1000 U/ml catalase. After forty-eight hours, cell numbers of each dish were counted by an automatic analyzer CountStar. All experiments were replicated a minimum of three times. Data are presented as means ± S.D. and statistical analysis was carried out using one-way ANOVA with Tukey's multiple comparison test (*$p<0.05$ versus control).
